# Editorial: Epigenetic modifications and cardiovascular diseases

**DOI:** 10.3389/fphar.2026.1870690

**Published:** 2026-05-14

**Authors:** Indira Pokkunuri, Cerrone R. Foster, Suman Dalal

**Affiliations:** 1 Department of Natural Sciences, University of Houston Downtown, Houston, TX, United States; 2 Department of Biological Sciences, East Tennessee State University, Johnson City, TN, United States; 3 Department of Biomedical Health Sciences, East Tennessee State University, Johnson City, TN, United States

**Keywords:** cardiovascular diseases, DNA methylation, epigenetics, extracellular vesicles, vascular remodeling

## Introduction

Cardiovascular disease (CVD) continues to impose economic and clinical burden on the healthcare system, and our ability to measure, predict or reverse maladaptive cardiac remodeling remains limited. Despite advances in biologic agents, diagnostic techniques, novel drugs, advanced imaging, and personalized approaches, our capacity to prevent or reverse CVD remodeling and improve patients’ outcomes remains modest. The main reason for this limited progress is the poor understanding of the molecular mechanisms governing cardiac remodeling. Emerging evidence over the past decade indicates epigenetic modulation, especially vascular DNA hypermethylation, plays a pivotal role in chromatin remodeling, gene expression, and manifestation of cardiac disease. While earlier studies reported hypomethylation, functional experiments revealed genome-wide, lesion-specific DNA hypermethylation as the defining epigenetic reprogramming within the vasculature. Cardiovascular risk factors, such as a high-fat diet, obesity, and diabetes increase DNA methyltransferase (DNMT) activity and/or hypermethylation, establishing it as a significant upstream event in disease progression.

Vascular cell types, including smooth muscle cells, endothelial cells, and macrophages, exhibit distinct epigenetic patterns that define their role in the pathology of CVDs, including atherosclerosis. For example, in the endothelial cells, disturbed blood flow induces DNA hypermethylation, increasing endothelial inflammation and atheroma formation at vascular sites. In her review, Zaina highlights the emerging potential of anti-DNMT drugs as therapeutic options for CVDs (Zaina), noting that inhibition of DNA methylation can reduce inflammation and attenuate atherosclerosis in animal models. Advanced drug delivery systems, such as nanotechnology, can deliver DNMT inhibitors directly to vascular walls, enabling localized modification of aberrant DNA methylations, ameliorating inflammation and atherosclerosis.

Vascular aging, driven by vascular smooth muscle cell senescence, leads to chronic inflammation and high mortality associated with CVD progression. Endogenous sulfur dioxide (SO2) is a newly identified key cardiovascular signaling mediator via epigenetic modifications in vascular smooth muscle cells (VSMC) acting as an intrinsic vasoprotective agent or as an age-related disease when absent. Huang et al. describe that the inhibition of interferon regulatory factor −1 (IRF-1), a pro-senescence-transcription factor, by SO2 reduces vascular inflammation and VSMC senescence by downregulating IL-1β, IL-6, p21 Cip/Waf within the arterial wall (Qiu et al.). While direct studies linking SO2 to DNA/Histone modifications are sparse, an emerging role for SO2 and sulfur metabolism-dependent regulation of transcription factors such as STAT3 suggests important indirect epigenetic effects. Interestingly, IRF1 dynamically interacts with differentially modified histones at promoter regions, influencing chromatin remodeling. These findings suggest that SO2 may exert its protective effect, potentially by epigenetically modulating gene expression in vascular smooth cells and identifying the SO2/IRF1 axis as a potential therapeutic strategy to rejuvenate the vasculature.

A common feature of all cardiac cell types in CVD is the necessity for balanced intercellular communication by extracellular vesicles (EVs). EVs carrying non-coding RNAs have emerged as key mediators of epigenetic regulation via intercellular signaling among cardiac cells in CVD. EVs derived from donor tissues and circulating sources target recipient cells, altering their biological functions, thereby impacting the progression of CVDs. Review by Hu et al. reveals how EV-associated non-coding RNAs contribute to cardiac hypertrophy, a specific type of cardiac remodeling (Qiu et al.). Notably, microRNAs function as key epigenetic regulators, modulating gene expression at the post-transcriptional level. By transporting these epigenetic regulators, EVs facilitate propagation of epigenetic signals between cells, thereby playing a significant role in epigenetically regulated pathological remodeling, including cardiac hypertrophy, inflammation, fibrosis, and angiogenesis.

In contrast, Sun et al. broaden the scope beyond microRNA to include EV-associated long non-coding RNAs and circular RNAs and examine their regulatory role in myocardial infarction, heart failure, atherosclerosis, and ischemia reperfusion injury (Gan et al.). The degree of EV secretion depends on the microenvironment and external cues, including cellular stress and activation signals. Therefore, identifying the cargo within the EVs may reflect how the transitioning state of their original cells could potentially serve as a biomarker for CVD. Taken together, the reviews by Hu and Gan highlight an emerging paradigm in which EV-associated non-coding RNA functions as a modular signaling system that coordinates multicellular responses during cardiac injury. Beyond advancing our mechanistic understanding of cardiac remodeling, these studies suggest that EV cargo may serve as circulating biomarkers to capture the dynamic state of cardiac tissues and underscore the therapeutic significance of EVs in CVD. However, standardization of EV isolation, cargo profiling, and *in vivo* validation of the EV will be crucial to enable clinical translation. Altogether, the convergence of RNA biology, EV, and cardiac remodeling presents an emerging frontier for biomarker discovery and therapeutic innovation.

Clinical application of these epigenetic markers in CVD is an important next step. Here, Ding et al. present a case report of a patient with pulmonary arterial hypertension (PAH) and a rare digenic mutation in FLNA and MMACHC genes (Zhang et al.) associated with PAH. Often referred to as the “malignancy of the cardiovascular system”, studies show that epigenetic regulation in endothelial smooth muscle cells plays a critical role in vascular remodeling and disease progression in PAH. While FLNA and MMACHC mutations provide a structural basis for the disease, they may not explain the variability in clinical presentation or severity of PAH. FLNA directs cytoskeletal reorganization, which may impact DNA modifications via chromatin and transcription factor remodeling, and MMACHC impacts cobalamin metabolism and is closely linked to methylation mechanisms. Mutations of these genes may disrupt global DNA methylation patterns, in turn acting as a key mediator in relaying downstream effects. Here, the patient responded positively to PAH-associated pharmacotherapy, followed by a 2-year follow-up, suggesting promising novel treatment strategies for PAH.

## Conclusion

These recent articles provide critical insights into the mechanisms of SO2 signaling, EV-mediated transfer of non-coding RNAs, mutations in key genes regulating DNA methylation, emphasizing how epigenetic modifications can be propagated in cells, resulting in amplified CVD progression. Collectively, they bridge genetic susceptibility with dynamic cellular responses, offering a more integrated understanding of cardiovascular pathology. Importantly, these studies identify novel therapeutic targets, including DNMTs and PAH pharmacotherapy, with the potential to improve patient outcomes.

